# Right Cu_2−_ *_x_*S@MnS Core–Shell Nanoparticles as a Photo/H_2_O_2_‐Responsive Platform for Effective Cancer Theranostics

**DOI:** 10.1002/advs.201901461

**Published:** 2019-08-27

**Authors:** Xiaojuan Huang, Guoying Deng, Yong Han, Guizhu Yang, Rujia Zou, Zhiyuan Zhang, Shuyang Sun, Junqing Hu

**Affiliations:** ^1^ Department of Oral and Maxillofacial‐Head & Neck Oncology Ninth People's Hospital Shanghai Jiao Tong University School of Medicine Shanghai 200011 P. R. China; ^2^ National Clinical Research Center for Oral Diseases Shanghai 200011 P. R. China; ^3^ Shanghai Key Laboratory of Stomatology & Shanghai Research Institute of Stomatology Shanghai 200011 P. R. China; ^4^ Trauma Center of Shanghai General Hospital Shanghai Jiao Tong University School of Medicine Shanghai 201620 P. R. China; ^5^ State Key Laboratory for Modification of Chemical Fibers and Polymer Materials College of Materials Science and Engineering Donghua University Shanghai 201620 P. R. China; ^6^ College of Health Science and Environmental Engineering Shenzhen Technology University Shenzhen 518118 P. R. China

**Keywords:** cancer treatment, Cu_2_*_−x_*S@MnS, patient‐derived xenografts, photo/H_2_O_2_‐responsive platforms, tumor cell line‐derived xenografts

## Abstract

Stimuli‐responsive nanomedicines have become a recent research focus as a candidate for cancer treatment because of their effectiveness, sensibility, and minimal invasiveness. In this work, a novel nanosystem is developed based on Cu_2−_ *_x_*S@MnS core–shell nanoparticles (CSNPs) in which the Cu_2−_ *_x_*S core serves as a photosensitizer to generate hyperthermia and reactive oxygen species (ROS), and the MnS shell is used in H_2_O_2_‐responsive O_2_ production. Cu_2_ *_−x_*S@MnS CSNPs with an independent core and shell ratio are synthesized by a controllable hot‐injection method, resulting in an optimal photothermal (PT) effect with a PT conversion efficiency of up to 47.9%. An enhanced photodynamic (PD) effect also occurs in an H_2_O_2_ environment. More significantly, in vivo experiments demonstrate that Cu_2_ *_−x_*S@MnS CSNPs can mediate tumor shrinkage in both HeLa tumor cell line‐derived xenograft (CDX) and head and neck squamous cell carcinoma (HNSCC) patient‐derived xenograft (PDX) models, with the capability of being used as a T1‐enhanced magnetic resonance (MR) contrast agent. These results suggest the great potential of as‐prepared Cu_2_ *_−x_*S@MnS CSNPs as photo/H_2_O_2_‐responsive therapeutic‐agents against tumors, even in a complicated and heterogeneous environment, thus promoting the clinical translation of nanomedicine.

## Graphical abstract

**Cu_2_*_−x_*S@MnS core–shell nanoparticles** are developed for cancer theranostics. The Cu_2_ *_−x_*S core serves as a photosensitizer to generate hyperthermia and reactive oxygen species, and the MnS shell is used in H_2_O_2_‐responsive O_2_ production, therefore mediating tumor shrinkage in both HeLa tumor cell line‐derived xenograft and head and neck squamous cell carcinoma patient‐derived xenograft models, with the capability of being used as a T1‐enhanced magnetic resonance contrast agent.

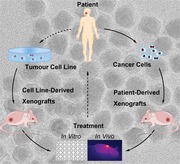

## 1 Introduction

Phototherapy has been heavily researched as a promising treatment option for cancer treatment due to its minimal invasiveness, high selectivity, and lower systemic toxicity.^1^ Until now, various nanoagents made of noble metals,^2^, ^3^ carbon‐based materials,^4^, ^5^ organic compounds,^6^, ^7^ and inorganic semiconductor compounds^8^, ^9^ have been proposed for cancer treatment upon photo‐activation. In particular, tumor microenvironment (TME)‐triggered nanostructures based on various manganese compounds conjugated with photosensitizers^10^, ^11^, ^12^ have attracted substantial attention. These nanostructures offer a unique type of nanotheranostics, which function in magnetic resonance imaging (MRI) and on‐demand toxic reactive oxygen species (ROS) production for photodynamic (PD) therapy upon photo‐excitation, as well as modulation of hypoxic TMEs to enhance the cancer therapy via H_2_O_2_‐response, thus resulting in comprehensive antitumor effects. However, most reported nanotheranostics are manganese‐supporting nanostructures with organic photosensitizers loaded and might not be ideal for realizing precisely controlled release of therapeutic payloads or supply of a sufficiently effective antitumor effect with mono‐therapy.

Similar to PD therapy, photothermal (PT) therapy is also a novel modality that operates upon appropriate laser irradiation, with negligible attenuation into biological tissues and minimal photodamage to cells of adjacent tissues.^13^, ^14^ The generated hyperthermia in PT therapy can selectively kill cancer cells via apoptosis/necrosis and alter the TME through increased vascular permeability and improved oxygen pressure levels, thus enhancing the tumor sensitivity to PD therapy.^15^, ^16^ The inhibition of DNA‐damage repair induced by ROS following hyperthermia further improves the PD treatment effect.^17^, ^18^ Copper sulphide, with numerous copper‐deficient stoichiometries (Cu_2_ *_−x_*S), is a promising PT agent due to its stoichiometry‐dependent near‐infrared region (NIR) localized surface plasmon resonance (LSPR) absorption derived from the copper vacancy.^8^, ^19^, ^20^ Moreover, copper sulphide also has the ability to mediate PD therapy in which laser irradiation stimulates the generation of ROS to ablate cancer cells,^9^, ^21^ thus improving the therapeutic effect with the potential to benefit from the advantages of each treatment mode. Therefore, integration of plasmonic Cu_2_ *_−x_*S and magnetic manganese compounds into a single unit could be advantageous for efficient PT applications and a hyperthermia/O_2_‐enhanced PD effect, as well as avoidance of photobleaching and uncontrolled release of organic photosensitizers.

Even though with the goal of improving tumor oxygenation and enhancing cancer treatment, performance evaluation of these proposed nanomaterials is mostly based on tumor cell line‐derived xenograft (CDX) mouse models.^22^, ^23^ However, these results cannot accurately reflect the clinical application value of the nanomedicine because the CDX models have adapted to the in vitro environment and undergone irreversible genetic and biological changes.^24^ Indeed, the delivery, accumulation, and penetration of nanomedicines and the sensitivity, tolerability, and distribution of heat and ROS generated in the phototherapy process are likely to be affected by the complexity, heterogeneity, and especially TME in the primary human tumor tissue (e.g., interstitial pressure, extracellular matrix barrier).^25^, ^26^, ^27^ Compared with the CDX model, the patient‐derived xenograft (PDX) model preserves the intratumoral heterogeneity and histology from the primary human cancer tissues, as well as the regenerative TME, such as blood vessels, tumor stroma, fibroblasts, macrophages, and extracellular matrices, thus making it more reliable for preclinical assessments of nanomedicines.^28^, ^29^, ^30^

In the current work, we prepared multifunctional Cu_2_ *_−x_*S@MnS core–shell nanoparticles (CSNPs) as a photo/H_2_O_2_‐responsive platform for enhanced cancer theranostics. The Cu_2_ *_−x_*S@MnS CSNPs were synthesized in a one‐pot hot‐injection method and were subsequently surface modified. The incorporation of Mn in the CSNP was found to vary in distribution and transfer of charge carriers, resulting in tunable absorption spectra. The intense optical absorption from the CSNPs in the NIR led to the perfect PT conversion and IR thermal imaging property. At the same time, the MnS shell endows the Cu_2_ *_−x_*S@MnS CSNPs with the ability to be used as a T1‐MR contrast agent. Furthermore, the Cu_2_ *_−x_*S@MnS CSNPs possess a catalase‐like activity to function as an H_2_O_2_‐responsive platform for enhancing the PD effect in which the Cu_2_ *_−x_*S serves as a photosensitizer. Above all, the catalase‐like Cu_2_ *_−x_*S@MnS CSNPs integrate PT therapy, enhanced PD therapy and MRI as a whole, thus demonstrating their potential against tumors in both CDX and PDX models and promoting further applications of nanomedicines based on these nanostructures.

## 2 Results and Discussion

### 2.1 Preparation and Characterization of Cu_2_ *_−x_*S@MnS CSNPs

**Figure 1:**
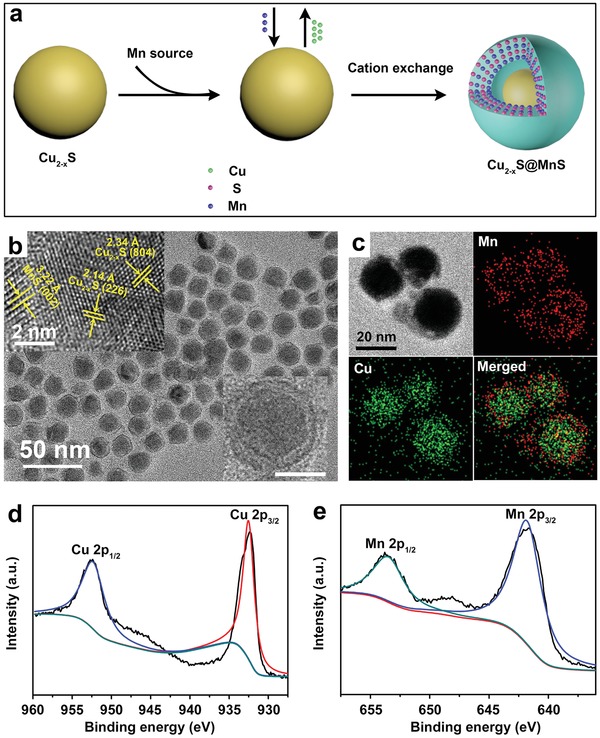
a) Synthesis scheme of Cu_2_ *_−x_*S@MnS CSNPs. b) TEM morphology of as‐synthesized CSNPs. Inset in the upper left corner: high‐resolution images. Inset in the lower right corner: TEM image of a single CSNP, scale bar = 10 nm. c) EDS elemental mapping of Cu_2_ *_−x_*S@MnS CSNPs. d,e) XPS spectra for Cu 2p and Mn 2p, respectively.

Cu_2_ *_−x_*S@MnS CSNPs were synthesized via a one‐pot hot‐injection method in an oleyamine environment under an N_2_ atmosphere. **Figure** **1**a offers a brief illustration of the synthetic process for the nanocomposites. First, copper acetate (Cu(CH_3_COO)_2_) was selected as the copper source to create the Cu_2_ *_−x_*S nanoseeds with the aid of 1‐dodecanethiol (C_12_H_25_SH).^31^, ^32^ The import process of Mn and formation of the Cu_2_ *_−x_*S@MnS CSNPs were conducted by injecting an oleylamine dispersion of manganese acetate (Mn(CH_3_COO)_2_·4H_2_O) into the vessel and reacting at 200–220 °C for 20 min.

As shown in Figure 1b, transmission electron microscopy (TEM) revealed that the final products demonstrated a uniform core–shell structure and a mono‐disperse state, and their phase and composition constituents were assessed using X‐ray diffraction (XRD) and X‐ray photoelectron spectroscopy (XPS). As shown in Figure S1 in the Supporting Information, the XRD patterns of the final products match well with the standard power diffraction pattern of monoclinic Cu_2_ *_−x_*S (JCPDS No. 23‐0959) and hexagonal MnS (JCPDS No. 03‐1062). In parallel, the high‐resolution transmission electron microscopy (HRTEM) image confirmed the lattice fringes from the core corresponding to the (804) and (226) planes of the Cu_2_ *_−x_*S crystal, with spacings of 2.34 and 2.14 Å, respectively. The lattice spacing of 3.22 Å in the shell is associated with the (002) plane of the MnS crystal. In the XPS data (Figure 1d,e, and Figure S2, Supporting Information), the peaks located at 952.5 and 932.6 eV respectively correspond to Cu 2p1/2 and Cu 2p3/2 levels, certifying the coexistence of Cu^+^ and Cu^2+^.^33^, ^34^ The characteristic peaks of 653.6 and 641.9 eV respectively accord with the Mn(II) 2p1/2 and Mn(II) 2p3/2 levels.^35^ Moreover, we conducted composition analysis of the CSNPs via TEM elemental mapping with copper and manganese windows (Figure 1c, sulphur window mapping in Figure S3 in the Supporting Information), further demonstrating that the MnS shell is independently located outside of the Cu_2_ *_−x_*S core.

**Figure 2:**
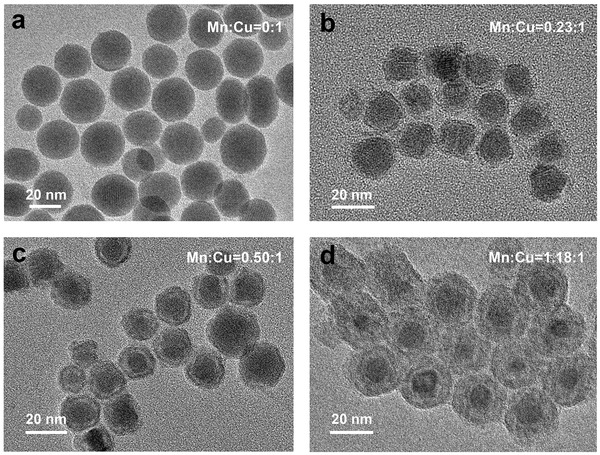
TEM images of a) pure Cu_2_ *_−x_*S NPs, b) thin‐shell Cu_2_ *_−x_*S@MnS CSNPs, c) intermediate‐shell Cu_2_ *_−x_*S@MnS CSNPs, and d) thick‐shell Cu_2_ *_−x_*S@MnS CSNPs.

The high chalcocite Cu_2_ *_−x_*S could be viewed as a solid–liquid hybrid phase with Cu ions diffusing between different sites, similar to a liquid,^36^ making the cation exchange reaction possible. Therefore, the growth of the Cu_2_ *_−x_*S@MnS CSNPs in our work includes nucleation of Cu_2_ *_−x_*S through the reaction of Cu and S sources, followed by partial transformation from Cu_2_ *_−x_*S to MnS via cation exchange.^37^, ^38^ To further understand the mechanism, we carefully studied the formation process by adjusting the reaction conditions after the manganese source injection. Initially, pure Cu_2_ *_−x_*S NPs (**Figure** **2**a) were formed without the manganese source. After Mn(CH_3_COO)_2_·4H_2_O in oleylamine was injected, a portion of the Cu in the Cu_2_ *_−x_*S nanoseeds was replaced by Mn, and the Cu_2_ *_−x_*S@MnS CSNPs with various shell thickness formed at different rates of temperature increase in the reaction process. As shown in Figure 2b, when the rate of temperature increase was maintained at 0.25 °C min^−1^, a thin layer of MnS shell was coated outside the Cu_2_ *_−x_*S core (thin‐shell Cu_2_ *_−x_*S@MnS). With the rates increased up to 0.55 and 1.00 °C min^−1^, intermediate‐shell Cu_2_ *_−x_*S@MnS (Figure 2c) and thick‐shell Cu_2_ *_−x_*S@MnS (Figure 2d) were formed, respectively, accompanied by diminishing Cu_2_ *_−x_*S size. The molar ratios of Mn to Cu in the above four NPs were 0:1, 0.23:1, 0.5:1, and 1.18:1, respectively, as determined by inductively coupled plasma atomic emission spectroscopy (ICP‐AES). Moreover, as the cation exchange proceeded, a void emerged between the MnS shell and the Cu_2_ *_−x_*S nucleus. It is believed that this unique phenomenon can be attributed to the unequal ion diffusion between Cu^+^ and Mn^2+^, where the more rapidly exported Cu^+^ diffusion process induced the nanoscale Kirkendall effect.^39^, ^40^

### 2.2 PT Properties of Cu_2_ *_−x_*S@MnS CSNPs

El‐Sayed and coworkers reported that an optimal PT conversion effect could be reached when the LSPR from the plasmonic NPs resonates with the incident light, which means that a stronger absorption of the incident light leads to a higher PT activity.^20^, ^41^ Therefore, the vis–NIR absorption spectra of the Cu_2_ *_−x_*S@MnS NPs with different MnS shell thicknesses were examined. The pure Cu_2_ *_−x_*S NPs have an optical absorption band centered at 1700 nm (Figure S4, Supporting Information), consistent with the high chalcocite Cu_2_ *_−x_*S material. After partial transformation to a MnS shell, the absorption band gradually blueshifted with an obvious intensification and moved to 950 nm for the thick‐shell Cu_2_ *_−x_*S@MnS CSNPs, indicating the strong interaction between the Cu_2_ *_−x_*S core and the MnS shell.

**Figure 3:**
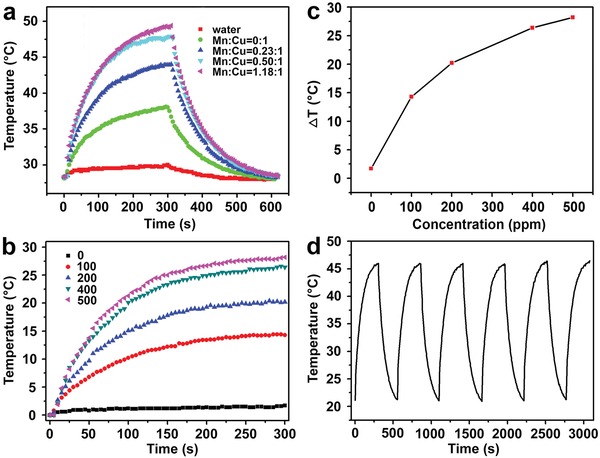
a) Temperature change curves of water and aqueous dispersions containing Cu_2_ *_−x_*S@MnS NPs (200 ppm) with different MnS shell thicknesses upon 808 nm laser irradiation (0.72 W cm^−2^, 5 min) and a subsequent cooling process. b) Temperature elevation curves of aqueous dispersions containing thick‐shell Cu_2_ *_−x_*S@MnS CSNPs with different concentrations under laser irradiation. c) Plot of temperature change (Δ*T*) versus concentration of thick‐shell Cu_2_ *_−x_*S@MnS CSNPs. d) Temperature change curve of thick‐shell Cu_2_ *_−x_*S@MnS suspension over laser on/off cycles.

Before comparison of their PT properties, the four as‐synthesized Cu_2_ *_−x_*S@MnS samples were first transferred from the organic phase into water using an amphiphilic poly(maleic anhydride)‐based polymer as a protecting group for the hydrophilic carboxylic acid groups and hydrophobic oleylamine sidechains. The Fourier transform infrared spectroscopy (FTIR) patterns (Figure S5, Supporting Information) showed that selected new peaks (at 1697, 1638, and 1210 cm^−1^) obviously appeared after material modification, representing the characteristic absorption peaks of C=O and C—O, which indicated successful hydrophilic modification. The PT conversion performances of these Cu_2_ *_−x_*S@MnS NPs were measured in aqueous dispersion under irradiation with an 808 nm laser for 5 min at an intensity of 0.72 W cm^−2^. **Figure** **3**a shows the temperature change curves. The temperature of the pure Cu_2_ *_−x_*S NPs suspension increased from 28.1 to 38.1 °C within 5 min. The thin‐shell Cu_2_ *_−x_*S@MnS and intermediate‐shell Cu_2_ *_−x_*S@MnS CSNPs suspensions had final temperatures of 44 and 47.9 °C, respectively, under the same conditions. The highest temperature of 49.4 °C after irradiation was stimulated by the thick‐shell Cu_2_ *_−x_*S@MnS CSNPs aqueous dispersion. In other words, the thick‐shell Cu_2_ *_−x_*S@MnS CSNPs obtained an optimal PT conversion efficiency upon photo‐excitation at 808 nm, and therefore, they were selected as the object for the following study.

To further investigate the PT property of our selected Cu_2_ *_−x_*S@MnS CSNPs, aqueous dispersions with various concentrations (0, 100, 200, 400, and 500 ppm) were excited by an 808 nm laser. The temperature elevation of these dispersions was found to be proportional to the Cu_2_ *_−x_*S@MnS concentration (Figure 3b,c). A concentration of 100 ppm showed a temperature increase of 14.3 °C after 5 min of irradiation. As the concentration increased to 500 ppm, the temperature increased by 28.2 °C. In comparison, the temperature of distilled water increased by only 2 °C. The PT conversion efficiency was calculated as 47.9% (Figure S6, Supporting Information) using a modified method, according to previous studies.^20^, ^42^ In addition, the laser on–off circulation tests displayed in Figure 3d demonstrate the stability of the thick‐shell Cu_2_ *_−x_*S@MnS CSNPs under photo‐excitation.

### 2.3 In Vitro O_2_ and ROS Generation of Cu_2_ *_−x_*S@MnS CSNPs

**Figure 4:**
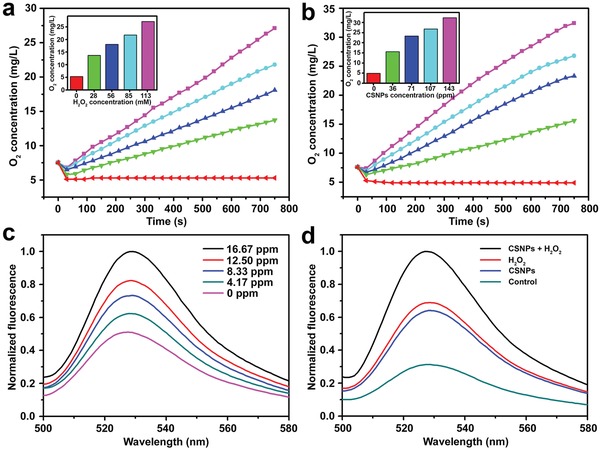
a) Dissolved oxygen concentration in aqueous dispersions containing Cu_2_ *_−x_*S@MnS CSNPs (71 ppm) with different H_2_O_2_ concentrations versus time. Insert: final dissolved oxygen concentration. b) Dissolved oxygen concentration in H_2_O_2_ solutions (71 × 10^−3^ m) with different Cu_2_ *_−x_*S@MnS CSNPs concentrations versus time. Insert: final dissolved oxygen concentration. c) Normalized fluorescence emission spectrum of SOSG (2.5 × 10^−6^ m) incubated with Cu_2_ *_−x_*S@MnS CSNPs suspensions (i.e., 0, 4.17, 8.33, 12.50, and 16.67 ppm) after 808 nm laser irradiation (0.72 W cm^−2^). d) Normalized fluorescence emission spectrum of SOSG with different treatments after laser irradiation.

As elements with multiple redox states, both copper and manganese show striking redox properties, similar to iron, which decomposes H_2_O_2_ through Fenton‐like pathways.^43^ Thus, substantial attention is focused on the peroxidase‐like activity of Cu_2_ *_−x_*S and the modulation of the hypoxic TME induced by manganese‐supporting nanostructures. Herein, we quantified the generation of O_2_ triggered by the thick‐shell Cu_2_ *_−x_*S@MnS CSNPs when exposed to H_2_O_2_ using a dissolved oxygen meter. As shown in **Figure** **4**a, without H_2_O_2_, the Cu_2_ *_−x_*S@MnS CSNPs suspension (71 ppm) maintained a low dissolved oxygen level. After addition of H_2_O_2_ (28 × 10^−3^ m) to the Cu_2_ *_−x_*S@MnS CSNPs suspension, a large amount of bubbles was observed, and the dissolved oxygen concentration increased from 7.6 to 13.7 mg L^−1^ within 12.5 min. Moreover, the dissolved oxygen concentration increased more rapidly and appeared to be higher post‐H_2_O_2_ addition with comparatively more H_2_O_2_ (56, 85, and 113 × 10^−3^ m, in Figure 4a) added or a higher concentration of Cu_2_ *_−x_*S@MnS CSNPs (107 and 143 ppm, in Figure 4b), indicating that additional O_2_ is generated from the oxidation of H_2_O_2_ induced by the Cu_2_ *_−x_*S@MnS CSNPs. However, for dispersions lacking Cu_2_ *_−x_*S@MnS CSNPs (C_CSNPs_ = 0, in Figure 4b), the dissolved oxygen concentrations remained constantly at a lower level, further confirming the necessity of the Cu_2_ *_−x_*S@MnS CSNPs for generation of O_2_.

Apart from their role as a PT agent, copper sulphides can also stimulate generation of ROS under laser irradiation.^9^, ^44^ Therefore, we assessed the generation of ROS induced by the thick‐shell Cu_2_ *_−x_*S@MnS CSNPs with singlet oxygen sensor green (SOSG), a fluorescence indicator for ^1^O_2_. As shown in Figure 4c, the aqueous dispersion containing SOSG exhibited weak fluorescence. The fluorescence intensity of SOSG at 525 nm obviously increased with Cu_2_ *_−x_*S@MnS CSNPs incubation under irradiation using an 808 nm laser. A larger concentration of the Cu_2_ *_−x_*S@MnS CSNPs suspension resulted in an obvious increase in the fluorescence intensity of SOSG. Furthermore, considering the dependence of ROS generation on O_2_, the generation of ROS upon H_2_O_2_ treatment was also assessed (Figure 4d). The fluorescence intensity of SOSG in the Cu_2_ *_−x_*S@MnS CSNPs suspension with H_2_O_2_ at 525 nm increased to 305.22% of that in control group after laser irradiation and was much larger than that with only Cu_2_ *_−x_*S@MnS CSNPs or H_2_O_2_ added under the same conditions. These results show that Cu_2_ *_−x_*S@MnS CSNPs can enhance the generation of ROS in response to the addition of H_2_O_2_ upon laser irradiation.

### 2.4 In Vitro Cytotoxicity of Cu_2_ *_−x_*S@MnS CSNPs

**Figure 5:**
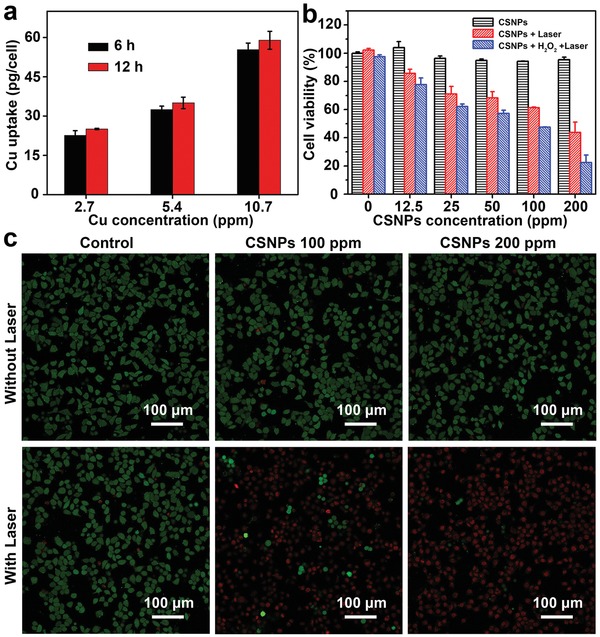
a) Concentration‐dependent cellular uptake of Cu determined by ICP‐AES after treatment with Cu_2_ *_−x_*S@MnS CSNPs suspensions for 6 and 12 h. b) Relative viabilities of tumor cells after incubation with Cu_2_ *_−x_*S@MnS CSNPs with or without addition of H_2_O_2_ and 808 nm light irradiation (0.72 W cm^−2^, 7 min). c) CLSM images of calcein AM/PI‐stained HeLa cells after different treatments.

Prior to assessing the capacity of the Cu_2_ *_−x_*S@MnS CSNPs to mediate cytotoxicity, we first analyzed their cellular uptake and internalization using HeLa cells. For visualization, the Cu_2_ *_−x_*S@MnS CSNPs were labeled with fluorescein isothiocyanate (FITC, excitation/emission at 494/525 nm) via covalent bonds. Cells were stained by 4,6‐diamidino‐2‐phenylindole (DAPI) and imaged with a confocal laser scanning microscope (CLSM) after incubation with the Cu_2_ *_−x_*S@MnS CSNPs. As shown in Figure S7 in the Supporting Information, a green signal appeared around the domains covered by DAPI after 20 min, suggesting that the CSNPs were taken up by cellular endocytosis and delivered to the cytoplasm. Moreover, we used ICP‐AES to quantize the NPs in the cells after incubation with Cu_2_ *_−x_*S@MnS CSNPs at different concentrations. With the increasing of CSNPs' concentration (**Figure** **5**a), the cellular uptake expressed by the copper content increased, and the internalized CSNPs had a higher amount at 12 h than that at 6 h.

The CCK‐8 assay was performed to evaluate the potential cytotoxicity of the Cu_2_ *_−x_*S@MnS material after HeLa cells were incubated with the CSNPs for 24 h (Figure 5b). Without NIR irradiation, the Cu_2_ *_−x_*S@MnS CSNPs suspension with concentrations ranging from 0 to 200 ppm showed negligible cytotoxicity and exhibited excellent biocompatibility, i.e., all cells retained greater than 90% viability. After 808 nm laser irradiation for 7 min, the Cu_2_ *_−x_*S@MnS CSNPs showed significant dose‐dependent cytotoxicity. Cells treated with only laser irradiation had a cell viability of nearly 100%. However, enhanced cytotoxicity occurred with Cu_2_ *_−x_*S@MnS CSNPs suspension concentrations of up to 100 and 200 ppm, with the cell viability decreasing to 61.39% and 43.82%, respectively. Additionally, similar results were achieved via live/dead cell costaining performed with calcein acetoxymethyl ester (calcein AM, green fluorescence) and propidium iodide (PI, red fluorescence). As shown in Figure 5c, no fluorescence changes were observed despite treatment of cells with laser irradiation or CSNPs at various concentrations. In contrast, obvious changes, with a dramatic increase in red color and decrease in green color, were observed in groups with both CSNPs and laser irradiation treatment, indicating significant dead cells. The cell‐killing effects of the Cu_2_ *_−x_*S@MnS CSNPs were greater when the suspension concentration increased from 100 to 200 ppm, confirming the photodamage of the Cu_2_ *_−x_*S@MnS CSNPs on cancer cells under 808 nm laser irradiation.

As verified above, the nanoplatforms based on Cu_2_ *_−x_*S@MnS CSNPs could mediate an efficient PT therapy effect under laser irradiation and generate O_2_ and ROS in response to H_2_O_2_. Moreover, it is known that the cancer cells inside solid tumors are able to constitutively produce H_2_O_2_ (from ≈50 × 10^−6^ to 100 × 10^−6^ m) by relying on the decreased antioxidant enzyme level. Therefore, Cu_2_ *_−x_*S@MnS CSNPs are anticipated to guide an enhanced phototherapy effect in response to H_2_O_2_ by generating O_2_ and more ROS after internalization by tumor cells. To confirm this, the in vitro cell cytotoxicity of Cu_2_ *_−x_*S@MnS CSNPs in an H_2_O_2_ environment was also evaluated via the standard CCK‐8 assay. HeLa cells were first incubated with Cu_2_ *_−x_*S@MnS CSNPs suspension for 24 h and subsequently treated with exogenous H_2_O_2_ (100 × 10^−3^ m). H_2_O_2_ did not influence cell viability under laser irradiation when the added CSNPs concentration was 0 (Figure 5b). Groups treated with both Cu_2_ *_−x_*S@MnS CSNPs and H_2_O_2_ exhibited comparatively more remarkable cell killing upon laser irradiation, with a cell viability (21.44%) lower than that in normoxic conditions at a CSNPs concentration of 200 ppm. These results indicated that extra H_2_O_2_ was helpful in enhancing the phototriggered cancer cell‐ablation efficiency of the Cu_2_ *_−x_*S@MnS CSNPs. Taken together, these results showed that Cu_2_ *_−x_*S@MnS CSNPs could mediate an improved therapeutic effect in response to H_2_O_2_ and photo‐irradiation.

### 2.5 MR Properties of Cu_2_ *_−x_*S@MnS CSNPs

**Figure 6:**
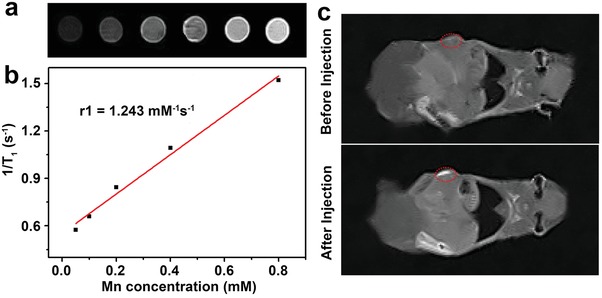
a) T1‐weighted MR images of Cu_2_ *_−x_*S@MnS CSNPs in aqueous solution at different Mn concentrations. b) Plots of the corresponding 1/T1 value of Cu_2_ *_−x_*S@MnS CSNPs as a function of Mn concentration. c) T1‐weighted MR images of mice before and after injection with Cu_2_ *_−x_*S@MnS CSNPs suspension.

Considering that manganese‐based nanomaterials are one of the most current T1‐enhanced MR contrast agents,^45^, ^46^ we explored the feasibility of using our thick‐shell Cu_2_ *_−x_*S@MnS CSNPs as an MR contrast agent. First, we recorded the MR images and relaxation times of aqueous dispersions containing Cu_2_ *_−x_*S@MnS CSNPs using a 0.5 T MR animal scanner (MesoMR23‐060H‐I) at varied Mn concentrations. As shown in **Figure** **6**a, the T1‐weighted MR images showed a brightening effect that was correlated with the Mn concentration and was further shown to be highly linear. According to the relaxation rate as a function of Mn concentration, the corresponding longitudinal relaxivity (*r*1, effectiveness as a contrast agent) of these CSNPs was calculated to be 1.243 mM^−1^ s^−1^, suggesting that our material is suitable for use as a contrast agent in MRI.

The contrast‐enhancing effect was evaluated in nude mice bearing HeLa tumors. Figure 6c shows the T1‐weighted MR images of the mouse before and after intratumoral injection of Cu_2_ *_−x_*S@MnS CSNPs dispersed in a saline solution. The tumor region has a contrast‐enhanced signal after CSNPs injection compared with that before, and the relative MR signal intensity in the tumor region shows an increase from 23 046 to 36 516 (Figure S8, Supporting Information). This result suggests the potential application of thick‐shell Cu_2_ *_−x_*S@MnS CSNPs as a promising MRI agent for tumor diagnosis.

### 2.6 In Vivo Tumor Shrinkage Efficacy of Cu_2_ *_−x_*S@MnS CSNPs in CDX Models

**Figure 7:**
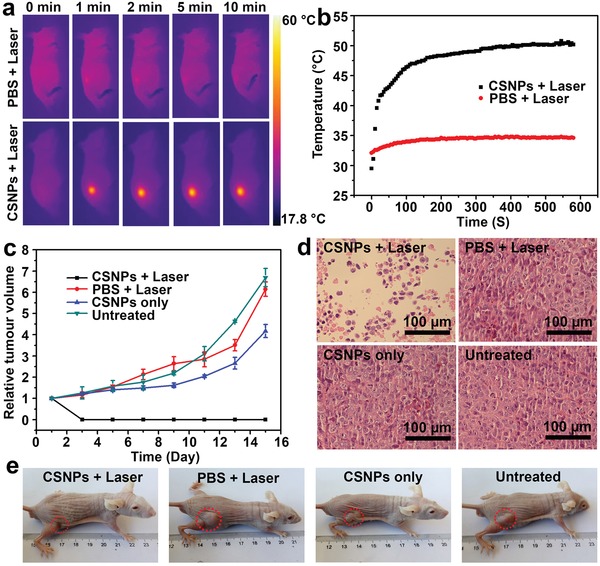
a) IR thermal images of HeLa tumor‐bearing mice in the CDX model with intratumoral injection of PBS and Cu_2_ *_−x_*S@MnS CSNPs suspension under 808 nm laser irradiation taken at different time intervals. The laser power density was 0.72 W cm^−2^. b) Temperature plots of the tumors in the two groups during the PT process. c) Average tumor sizes collected from mice of different groups after various treatments. d) Representative H&E‐stained histological images of ex vivo tumor sections. e) Representative photos of mice collected on the 16th day after treatments.

Based on the in vitro cytotoxicity of Cu_2_ *_−x_*S@MnS CSNPs upon laser irradiation, we assessed the in vivo phototherapy effect. CDX mice bearing HeLa tumors were first intratumorally injected with CSNPs suspension (100 µL, 200 ppm) and subsequently laser irradiated (808 nm, 0.72 W cm^−2^). The control groups were treated with PBS injection (100 µL) and the same laser exposure. A thermal infrared camera was used to record the full‐body IR thermal images and monitor the temperature changes in the mice, revealing massive contrast differences in tumor sites between the two groups (**Figure** **7**a). The temperature plots in Figure 7b further indicate that laser treatment caused the tumors of the CSNP‐bearing mice to rapidly increase to greater than 50 °C, which is known to be sufficiently high to induce damage in tumor cells.^47^ In contrast, the surface temperature of the tumor region of the PBS and laser‐treated mice increased by less than 3 °C under the same conditions, suggesting that Cu_2_ *_−x_*S@MnS CSNPs could efficiently absorb photo‐energy and convert it into local heat in vivo.

To investigate the potential antitumor efficacy of our thick‐shell Cu_2_ *_−x_*S@MnS CSNPs, tumors in mice were allowed to grow to a size of 5–10 mm in diameter, and the mice were randomly assigned to four groups (i.e., untreated, PBS + laser, CSNPs only, CSNPs + laser). We subsequently measured the body weight and tumor sizes of the mice during a 16 day period, starting from the laser irradiation on the first day. The results showed no significant difference in body weights between the treatment group (CSNPs + laser) and the other three control groups (Figure S9, Supporting Information), implying that the thick‐shell Cu_2_ *_−x_*S@MnS nanomaterials and laser irradiation showed no overall side effects in mice. Relative tumor volumes were calculated based on the size measured for each mouse. As shown in Figure 7c, mice in the control groups (untreated, PBS + laser, CSNPs only) all exhibited large increases in tumor size (respectively averaging 6.69‐, 6.14‐, and 4.17‐fold increases in relative volume on the 16th day), whereas the tumor sizes in the (CSNPs + laser)‐treated mice decreased. In addition to these obvious differences in tumor size as a result of phototherapy, we also noted that the tumors of the mice in the CSNPs + laser group turned black on the second day, again strongly indicating the rapid efficacy of our CSNPs‐mediated PT therapy. The representative photographs of these mice on the 16th day (Figure 7e) also confirmed that the mice showed tumor shrinkage only after CSNPs + laser treatment.

### 2.7 Antitumor Efficacy of Cu_2_ *_−x_*S@MnS CSNPs in PDX Models

The PDX model not only preserves the intratumoral heterogeneity and histology of the primary human cancer tissues but also preserves the regenerative TME, such as blood vessels, tumor stroma, fibroblasts, macrophages, and extracellular matrices, thus making it more significant for preclinical assessments of nanomedicines compared with CDX.^28^ Therefore, to further explore the clinical potential of phototherapy based on our thick‐shell Cu_2_ *_−x_*S@MnS CSNPs, a PDX model was constructed based on head and neck squamous cell carcinoma (HNSCC), which belongs to superficial tumor that is preferable for photo‐illumination and photo‐penetration. The 4th generation of PDX animals was used in this work due to its stabilization (Figure S10 in the Supporting Information shows the tumor volume change curves of mice in the first three generations). Similar to the experiments on the HeLa tumour CDX model (as shown in Figure S11 in the Supporting Information), mice inoculated with the PDX model were intratumorally injected with the CSNPs suspension (100 µL, 200 ppm) followed by laser irradiation (808 nm, 0.72 W cm^−2^) (Group I: CSNPs + laser, *n* = 6). Controls in these experiments included mice lacking CSNPs injection (Group II: PBS + laser, *n* = 6), mice lacking laser irradiation (Group III: CSNPs only, *n* = 6), and mice lacking both treatments (Group IV: untreated, *n* = 6).

**Figure 8:**
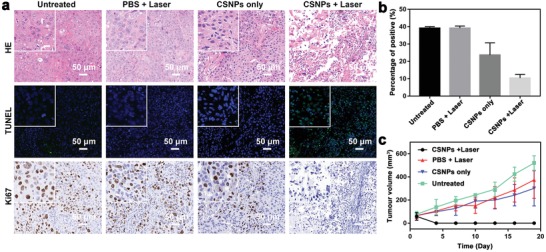
a) Representative H&E, TUNEL, and Ki‐67 stained images of ex vivo tumor sections after various treatments in the HNSCC PDX model. Insert: Images with high magnification, scale bar: 20 µm. b) Percentage of positive proliferating cells calculated from Ki‐67. c) Average tumor sizes collected from mice from different groups.

Over these treatments, we evaluated the therapeutic efficacy using haematoxylin and eosin (H&E) staining (**Figure** **8**a). Little damage was found in the tumor tissues from the groups treated with PBS + laser or CSNPs only compared with the untreated group, whereas evident cellular damage such as deformation, nuclei condensation, and destruction of membrane integrity was observed in the CSNPs + laser group. Moreover, to tentatively identify which cellular mechanisms are responsible for the cell death in tumors, we performed a terminal deoxynucleotidyl transferase dUTP nick end labeling (TUNEL) assay for apoptotic cell death and conducted immunofluorescence labeling using antibodies against a cell proliferation marker (Ki‐67). The TUNEL assay demonstrated that Cu_2_ *_−x_*S@MnS CSNPs induced the highest cell apoptosis rate in tumors upon laser excitation. A decrease in the number of Ki‐67‐positive proliferating cells was observed in tumors treated with CSNPs + laser (10% positive) compared with the other three groups (40% positive for blank control, 39% positive for PBS + laser control, and 25% positive for CSNP only control). These results suggested that Cu_2_ *_−x_*S@MnS CSNPs had an inhibitory effect on tumor cell proliferation and pro‐apoptosis. The decrease in positive proliferating cells in the CSNPs control might be due to the Fenton effect induced by the Cu_2_ *_−x_*S@MnS material within the special TME with a high H_2_O_2_ level. In addition, the tumor growth curves (Figure 8c) further confirmed the tumor shrinkage efficacy of Cu_2_ *_−x_*S@MnS CSNPs in the PDX models with the laser irradiation, and the bodyweight plots of mice (Figure S12, Supporting Information) proved the safety of phototherapy stimulated by CSNPs, thus reinforcing that our Cu_2_ *_−x_*S@MnS CSNPs can be used as an efficient therapeutic agent for cancer therapies.

### 2.8 In Vivo Biosafety of Cu_2_ *_−x_*S@MnS CSNPs

The in vivo biosafety of nanomedicines is always a considerable concern for application in cancer theranostics. To test biosafety, nude mice were used as models and intravenously injected with the Cu_2_ *_−x_*S@MnS CSNPs suspension. Blood and tissue samples were collected and analyzed at different time points. The results showed that Cu_2_ *_−x_*S@MnS material treatment did not affect the normal range of blood biochemistry indicators (Figure S13, Supporting Information) including alanine aminotransferase (ALT), aspartate transaminase (AST), blood urea nitrogen (BUN), creatinine (CRE), and uric acid (UA), and the physiological morphology of tissues including heart, liver, spleen, lung, and kidney, observed from H&E‐stained images in Figure S14 in the Supporting Information. Moreover, tissue samples were also digested with HNO_3_/HCl to determine the Cu contents via ICP‐AES measurements. As shown in Figure S15 in the Supporting Information, the Cu_2_ *_−x_*S@MnS material distribution reached a high level in liver and spleen at first, which could be possibly correlated with reticuloendothelial system (RES) uptake.^48^, ^49^ This distribution decreased significantly at 72 h post injection, indicating elimination from the organisms within 3 days. These preliminary investigations confirmed the biosafety of Cu_2_ *_−x_*S@MnS CSNPs at the tested dose. However, additional systematic studies of pharmacokinetics, pharmacodynamics and pharmaco‐immunology are still necessary for future clinical translation of such a material.

## 3 Conclusion

In conclusion, a novel nanosystem based on Cu_2_ *_−x_*S@MnS CSNPs was developed for cancer theranostics using a controllable hot‐injection method. The external MnS shell mediates O_2_ production to overcome hypoxia and regulates TME in a special tumor environment with excessive H_2_O_2_. The Cu_2_ *_−x_*S core is excited by a NIR laser, leading to an enhanced PD effect with an O_2_ self‐supplement condition and an improved PT effect via ROS and heat generation, resulting in successful eradication of subcutaneous tumors in CDX and PDX models. Moreover, the MR effect induced by the Mn‐based nanomaterial can be used to noninvasively locate tumors and guide treatment. This multifunctional nanoplatform demonstrated effective tumor ablation and imaging, promoting clinical translation of nanomedicine.

## 4 Experimental Section

*Materials and Reagents*: Copper (II) acetate (Cu(CH_3_COO)_2_), manganese (II) acetate (Mn(CH_3_COO)_2_·4H_2_O), sodium carbonate (Na_2_CO_3_), cyclohexane (C_6_H_12_), and chloroform (CHCl_3_) were obtained from Sinopharm Chemical Reagent Co. Ltd, and 1‐dodecanethiol (CH_3_(CH_2_)_11_SH) and oleylamine (OM) were sourced from Aladdin Industrial Corporation. Tetrahydrofuran (C_4_H_8_O) was purchased from Shanghai Boer Chemical Reagent Co. Ltd. Poly(isobutylene‐maleic anhydride) was obtained from Sigma‐Aldrich Chemie GmbH. Fluorescein isothiocyanate was obtained from Shanghai Macklin Biochemical Co. Ltd. All chemicals were of at least analytical reagent grade and used without further purification.

*Synthesis of Cu_2−x_S@MnS CSNPs*: The Cu_2_ *_−x_*S@MnS CSNPs were synthesized using a one‐pot hot‐injection method. In brief, 0.6 mmol Cu(CH_3_COO)_2_ was dissolved in 20 mL of oleylamine under vigorous stirring, and 8 mL of 1‐dodecanethiol was added. The mixture was heated to 220 °C in an N_2_ atmosphere and maintained for 15 min. Subsequently, 8 mL of oleylamine containing 1 mmol Mn(CH_3_COO)_2_·4H_2_O was injected into the hot solution, and the temperature of the solution was changed to 195 °C. The reactions continued for another 20 min with different rates of temperature increase (i.e., 0.25, 0.55, and 1.00 °C min^−1^). Finally, solid black products were collected by centrifugation after cooling to room temperature.

Pure Cu_2_ *_−x_*S NPs were prepared using the procedures above but without the addition of Mn(CH_3_COO)_2_·4H_2_O and following another 20 min reaction.

*Surface Modification of Hydrophobic Cu_2−x_S@MnS CSNPs*: The amphiphilic polymer composed of poly(maleic anhydride) and oleylamine was synthesized based on a protocol in the literature.^50^ For surface modification of hydrophobic Cu_2_ *_−x_*S@MnS CSNPs, 150 µL of amphiphilic polymer stock solution (0.8 m in CHCl_3_) and 10 mL of hydrophobic Cu_2_ *_−x_*S@MnS CSNPs (500 ppm in CHCl_3_) were combined and stirred for 4 h at room temperature. The solvent was removed by rotary evaporation, and 25 mL sodium carbonate solution (0.05 g mL^−1^) was added to the flask and stirred for 15 min at room temperature to disperse the hydrophilic Cu_2_ *_−x_*S@MnS CSNPs. The solution was centrifuged and dispersed into PBS for further use.

To prepare the hydrophilic Cu_2_ *_−x_*S@MnS‐FITC CSNPs, 5 mg of FITC was added into an amphiphilic polymer stock solution (20 mg) and stirred for 24 h in the dark until CHCl_3_ was volatilized. The hydrophobic Cu_2_ *_−x_*S@MnS CSNPs were modified with this production using the above procedures.

*In Vitro PT Performances of Cu_2−x_S@MnS CSNPs*: To measure the PT effect of Cu_2_ *_−x_*S@MnS CSNPs with different MnS shell thicknesses, 100 µL of aqueous dispersions containing pure Cu_2_ *_−x_*S NPs, thin‐shell Cu_2_ *_−x_*S@MnS CSNPs, intermediate‐shell Cu_2_ *_−x_*S@MnS CSNPs, and thick‐shell Cu_2_ *_−x_*S@MnS CSNPs were each irradiated by an 808 nm laser (Shanghai Xilong Optoelectronics Technology Co., Ltd., China) at a power density of 0.72 W cm^−2^. A thermal imaging camera (FLIR A300, USA) was used to record the temperature change.

To further investigate the PT effect of the selected thick‐shell Cu_2_ *_−x_*S@MnS CSNPs, 100 µL of aqueous dispersions with different concentrations (0–500 ppm) were irradiated using the 808 nm laser for 5 min. In the photostability test, the sample was laser irradiated for 5 min and cooled naturally. The on–off process was repeated six times.

*O_2_ Generation of Cu_2−x_S@MnS CSNPs in Response to H_2_O_2_*: A portable dissolved oxygen meter was used to measure O_2_ generation. First, the Cu_2_ *_−x_*S@MnS CSNPs were dispersed in 7 mL of aqueous solution with a concentration of 71 ppm, and different amounts of H_2_O_2_ were added. The dissolved oxygen concentration in the aqueous dispersions was recorded every 30 s after H_2_O_2_ was added. For investigation of the effect of the Cu_2_ *_−x_*S@MnS CSNPs suspension concentration on O_2_ release, the amount of H_2_O_2_ was added and maintained at 71 × 10^−3^ m, and the Cu_2_ *_−x_*S@MnS CSNPs concentration was changed from 0 to 143 ppm.

*ROS Production*: For ROS detection, the commercial chemical probe SOSG was used to detect its fluorescence intensity at 525 nm via fluorescence emission spectroscopy excited at 488 nm. SOSG (2.5 × 10^−6^ m) was added to 1 mL of Cu_2_ *_−x_*S@MnS CSNPs suspension (0–16.67 ppm), and the mixture was kept in the dark and irradiated with an 808 nm laser (0.72 W cm^−2^) for 60 min. Fluorescence emission spectroscopy was recorded. To test ROS production in response to H_2_O_2_, laser irradiation was conducted after addition of H_2_O_2_ (71 × 10^−3^ m).

*Cell Uptake*: HeLa cells were seeded into a 96‐well plate at 1 × 10^4^ cells per well at 37 °C in the presence of 5% CO_2_ for 24 h. The Cu_2_ *_−x_*S@MnS CSNPs suspensions were added to these wells at various concentrations (Cu concentrations were 10.709, 5.355, and 2.677 ppm, as measured by ICP‐AES). After incubation for 6 and 12 h, the medium was removed, each well was washed three times with PBS, and HeLa cells were digested with HNO_3_/HCl to determine the Cu uptakes via ICP‐AES measurements.

For visualization, HeLa cells were seeded into a 6‐well plate and cocultured with Cu_2_ *_−x_*S@MnS‐FITC CSNPs. The medium was removed, and each well was washed three times with PBS. The cell nuclei were stained with DAPI for 20 min. Finally, the cells were observed by CLSM. The Cu_2_ *_−x_*S@MnS‐FITC CSNPs and DAPI were excited separately using laser excitation at 494 and 358 nm and captured by emission at 525 and 461 nm.

*Biocompatibility of Cu_2−x_S@MnS CSNPs*: The in vitro cytotoxicity was evaluated using a CCK assay. HeLa cells were seeded into a 96‐well plate at 1 × 10^4^ cells per well at 37 °C in the presence of 5% CO_2_. After incubation for 24 h, the Cu_2_ *_−x_*S@MnS CSNPs suspensions were added to the wells at various concentrations and incubated for another 24 h. An amount of 200 µL complete medium with 10% (volume) CCK‐8 dispersion was added to each well of the microtiter plate and incubated in the CO_2_ incubator for another 2 h. The optical density (OD) was measured at 490 nm using a microplate reader. All experiments were independently repeated three times.

*In Vitro Phototherapy Effect*: HeLa cells were seeded into a 96‐well plate at 1 × 10^4^ cells per well at 37 °C in the presence of 5% CO_2_ for 24 h. The Cu_2_ *_−x_*S@MnS CSNPs suspensions were added to the wells at various concentrations. After 24 h, cells were irradiated with an 808 nm laser at 0.72 W cm^−2^ for 7 min. After further incubation for another 2 h, the cell viability was detected using a CCK assay and observed on a CLSM after costaining with calcein AM and PI for 20 min.

To study the effect of H_2_O_2_ on phototherapy, H_2_O_2_ (100 × 10^−3^ m) was added to the wells after Cu_2_ *_−x_*S@MnS CSNPs suspension addition, and the cell viability was detected using a CCK assay after laser irradiation.

*Animals and Tumor Model*: All animal experiments were performed in accordance with the guidelines of the Institutional Animal Care and Use Committee of Shanghai Jiao Tong University School of Medicine. For the CDX model, cancer cells from a HeLa tumor cell line were subcutaneously injected into BALB/c nude mice (6–8 weeks old).

For the PDX model, tumor samples were obtained from Shanghai Ninth People's Hospital with written informed consent from each patient and research ethics board approval in accordance with the Declaration of Helsinki. Herein, a tumor tissue sample from an HNSCC patient after surgical treatment was cut into ≈5 mm pieces and implanted into the BALB/c nude mice. After the tumor volume reached ≈1000 mm^3^, the mice were sacrificed, and the tumor tissues were sampled and implanted into another group of mice with the same protocol. The 4th generation of animals was used in in vivo experiments in this study.

*MR Measurement*: For in vitro MR measurement, Cu_2_ *_−x_*S@MnS CSNPs suspensions with different Cu concentrations were scanned under a 0.5 T small animal MR scanner (MesoMR23‐060H‐I) at room temperature. For MRI in vivo, HeLa tumor‐bearing mice were scanned with the same MR scanner before and after intratumour injection with Cu_2_ *_−x_*S@MnS CSNPs suspension (100 µL, 200 ppm). During the experiments, all mice were first anaesthetized using chloral hydrate (8%).

*In Vivo Therapy Experiment*: Tumors in mice were allowed to grow to a size of 5–10 mm in diameter and the mice were randomly assigned to four groups: Group I, untreated control; Group II, PBS + laser; Group III, CSNPs only; and Group IV, CSNPs + laser. For Groups III and IV, the Cu_2_ *_−x_*S@MnS CSNPs suspensions (100 µL, 200 ppm) were injected intratumorally. Mice in Groups II and IV were subsequently irradiated by an 808 nm laser at a power density of 0.72 W cm^−2^ for 10 min, and an infrared camera was applied to monitor the temperature changes. The tumor sizes were measured after treatments using a Vernier caliper. The tumor volume was calculated according to the following formula: volume = 0.5 × length × width^2^.

For tumor sectioning and staining experiments, the tumors from different groups were harvested 4 h after treatment, embedded in paraffin, and cryo‐sectioned into 4 µm slices using a conventional microtome. The slides were stained with H&E, TUNEL, and Ki‐67 and subsequently observed.

*In Vivo Systemic Toxicity Study*: Tumor‐bearing mice were intravenously injected with the Cu_2_ *_−x_*S@MnS CSNPs suspension (125 µL, 200 ppm). The control group was untreated. At different time points, major tissues including heart, liver, spleen, lung, and kidney were collected and digested with HNO_3_/HCl to determine the Cu contents by ICP‐AES measurements. Blood was collected for blood biochemistry analysis. Moreover, these organs were observed with H&E staining for comparison with untreated healthy mice.

## Conflict of Interest

The authors declare no conflict of interest.

## Supporting information

Supplementary
