# Supplementary material for: Right Cu2− xS@MnS Core–Shell Nanoparticles as a Photo/H2O2‐Responsive Platform for Effective Cancer Theranostics
Source: Adv Sci (Weinh). 2019 Aug 27;6(20):1901461. doi: 10.1002/advs.201901461 (PMC6794717; doi:10.1002/advs.201901461)
Supplement: Supplementary file 1 — Supplementary [file ADVS-6-1901461-s001.pdf]

Copyright WILEY-VCH Verlag GmbH & Co. KGaA, 69469 Weinheim, Germany, 2019.

ADVANCED SCIENCE
Open Access

Supporting Information

for *Adv. Sci.,* DOI: 10.1002/advs.201901461

Right $Cu_{2-x}S$@MnS Core–Shell Nanoparticles as a Photo/H_2O_2-Responsive Platform for Effective Cancer Theranostics

Xiaojuan Huang, Guoying Deng, Yong Han, Guizhu Yang, Rujia Zou, Zhiyuan Zhang, Shuyang Sun,* and Junqing Hu**

Copyright WILEY-VCH Verlag GmbH & Co. KGaA, 69469 Weinheim, Germany, 2019.

Supporting Information

Right $Cu_{2-x}S$@MnS core-shell nanoparticles as a photo/H_2O_2-responsive platform for effective cancer theranostics

Xiaojuan Huang, Guoying Deng, Yong Han, Guizhu Yang, Rujia Zou, Zhiyuan Zhang, Shuyang Sun*,* and *Junqing Hu**

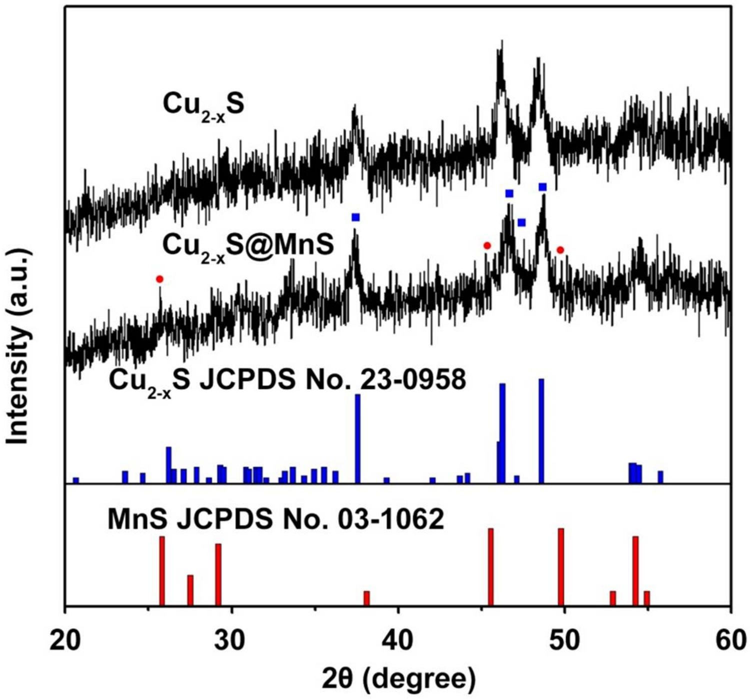

Figure S1. XRD pattern of as-prepared $Cu_{2-x}S$ NPs and $Cu_{2-x}S$@MnS CSNPs. The blue and red dots above the black line denote monoclinic $Cu_{2-x}S$ (JCPDS No. 23-0959) and hexagonal MnS (JCPDS No. 03-1062), respectively.

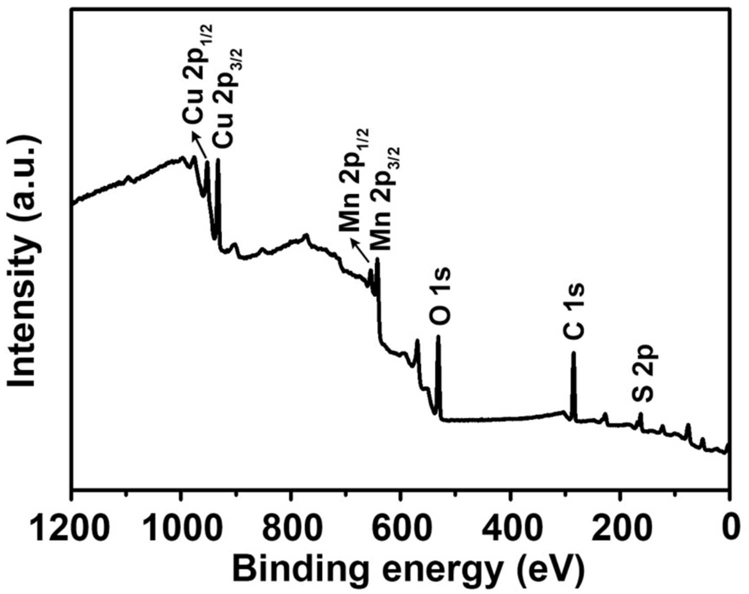

Figure S2. XPS survey spectrum of $Cu_{2-x}S$@MnS CSNPs.

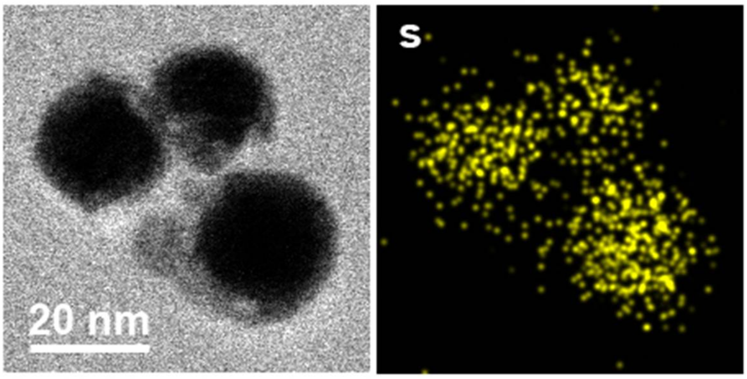

Figure S3. TEM elemental mapping with the sulphur window of $Cu_{2-x}S$@MnS CSNPs.

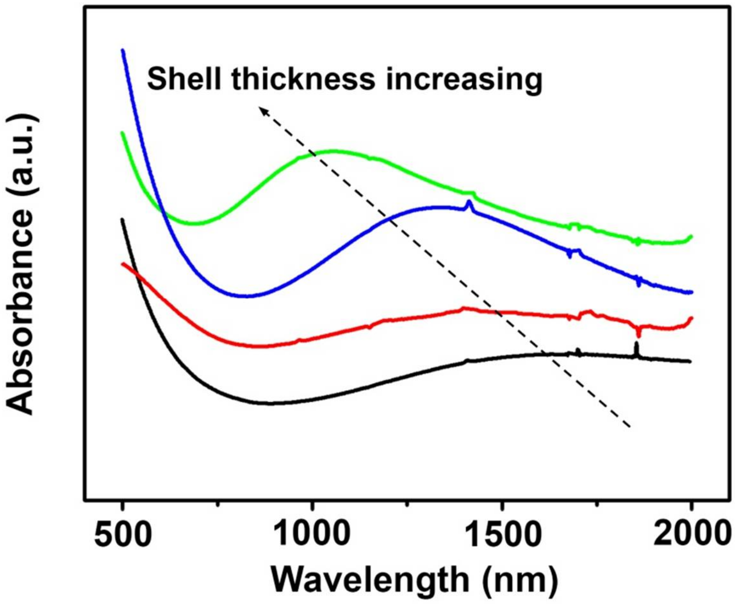

Figure S4. Vis-NIR absorption spectroscopy of the $Cu_{2-x}S$@MnS CSNPs with different MnS shell thicknesses.

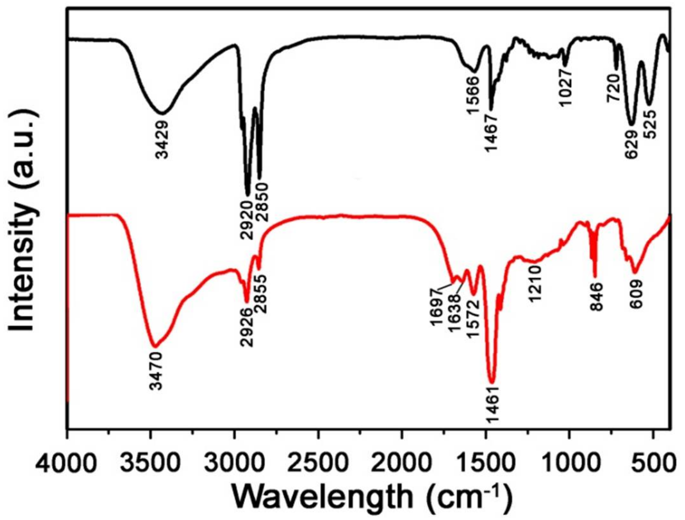

Figure S5. FTIR spectroscopy of $Cu_{2-x}S$@MnS CSNPs before and after surface modification.

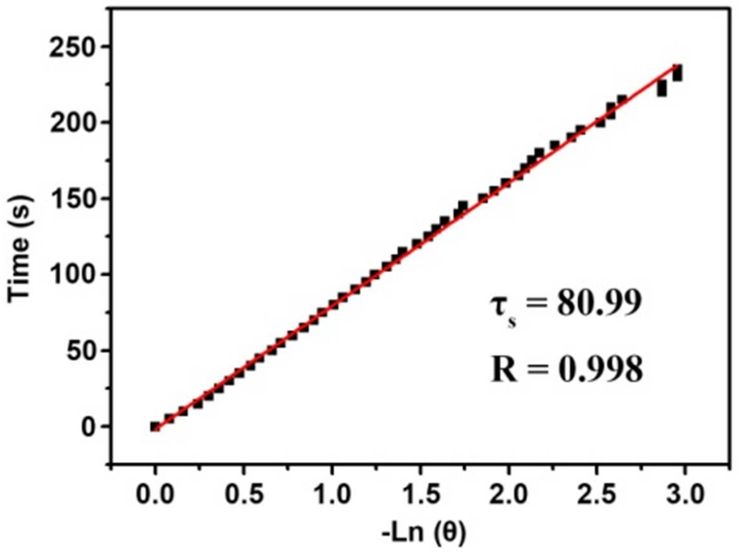

Figure S6. Time constant for heat transfer from the system (τ_s), calculated from the cooling period of the panel (Figure 3a).

The photothermal conversion efficiency (η) is calculated using the following equations, according to published studies from Roper and Hu et al.[1, 2]

$$\eta_T = \frac{hS(T_{max}-T_{surr})-Q_{dis}}{I(1-10^{-A_\lambda})} \quad (1)$$

$$hS = \frac{m \cdot C_{H2O}}{\tau_s} \quad (2)$$

$$t = -\tau_s ln\theta = -\tau_s \ln(\frac{T-T_{surr}}{T_{max}-T_{surr}}) \quad (3)$$

where h, S, and Q_{dis} are the respective heat transfer coefficient, irradiated area, and baseline energy inputted by the sample cell; T_{max} and T_{surr} are the highest temperature of the system and the surrounding temperature, respectively; I and $A_{808\ nm}$ are the respective power density of the laser and absorption of $Cu_{2-x}S$@MnS CSNPs at 808 nm; m and C_p are the mass and thermal capacity of the sample, respectively; and t is the time of the cooling process after irradiation.

In this work, τ_s was calculated as 80.99 s, as obtained from the linear fitting in Figure S6. The values of m and C_p are 0.1 g and 4.2 J/(g·□) , respectively, and Q_{dis}, A_{808nm}, I and (T_{max} - T_{surr}) are measured as 15.45 mW, 0.537, 276.48 mW, and 21.1 □, respectively. Above all, the photothermal conversion efficiency was calculated to be 47.9%.

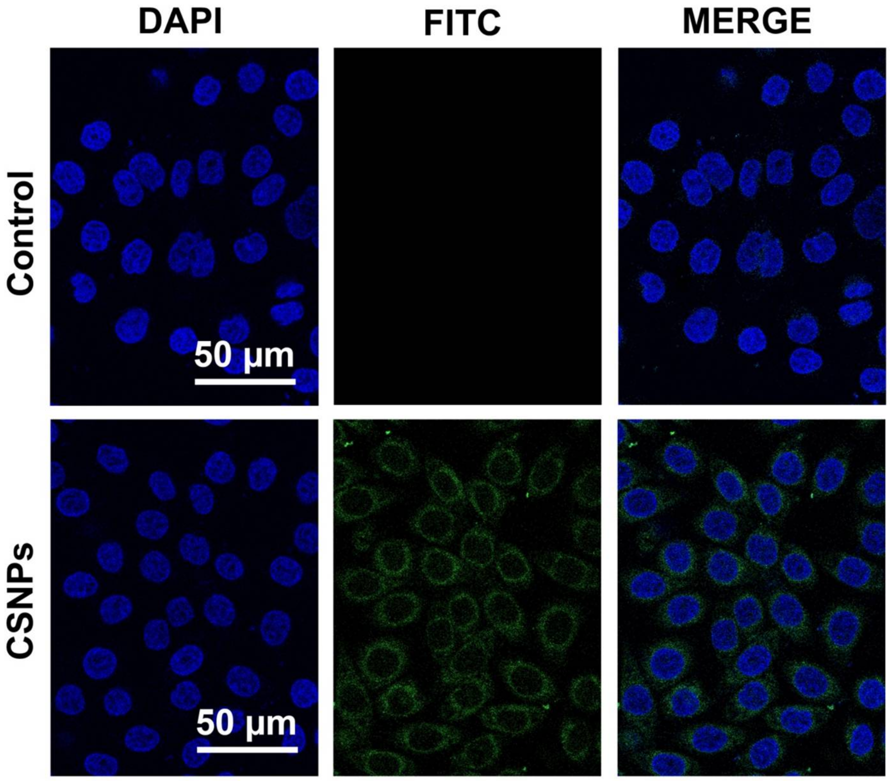

Figure S7. CLSM images of HeLa cells incubated with $Cu_{2-x}S$@MnS-FITC CSNPs suspension.

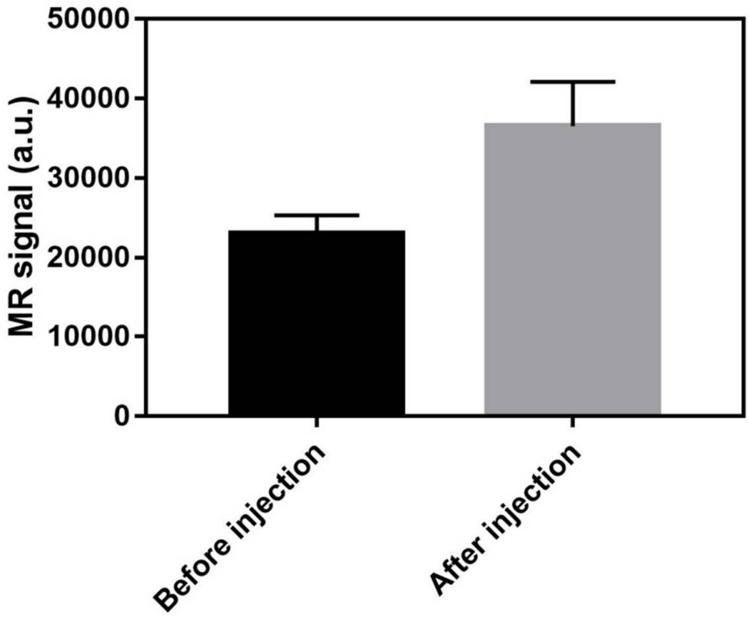

Figure S8. Relative MR signal intensity in tumour regions of mice before and after material injection.

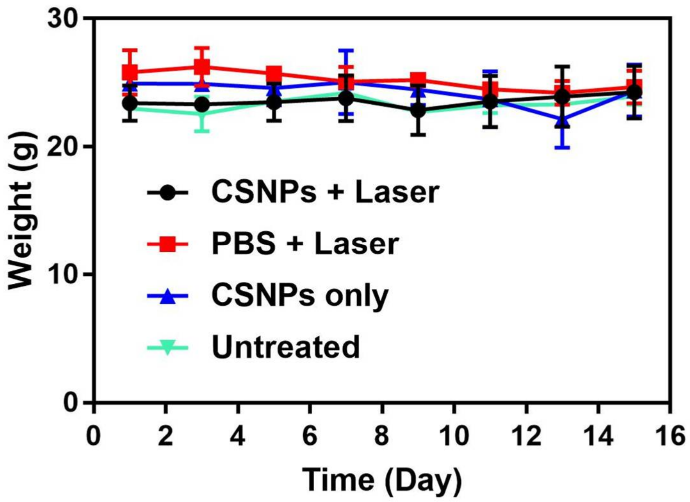

Figure S9. Average bodyweight curves of mice in different groups after various treatments in the CDX models.

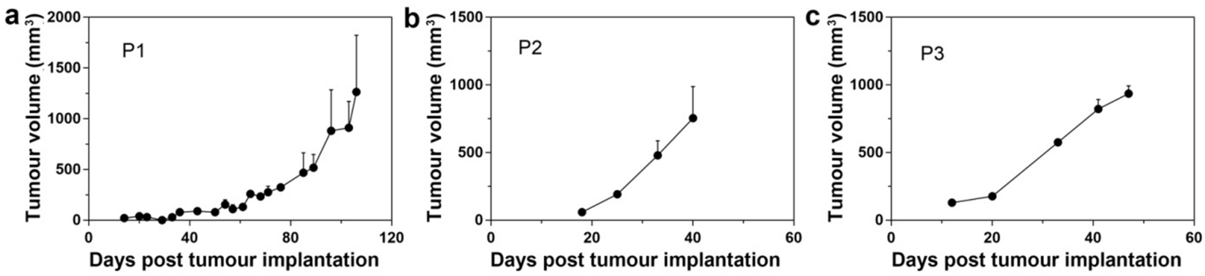

Figure S10. Tumour volume change curves of (a) P1, (b) P2, and (c) P3 mice in the PDX model.

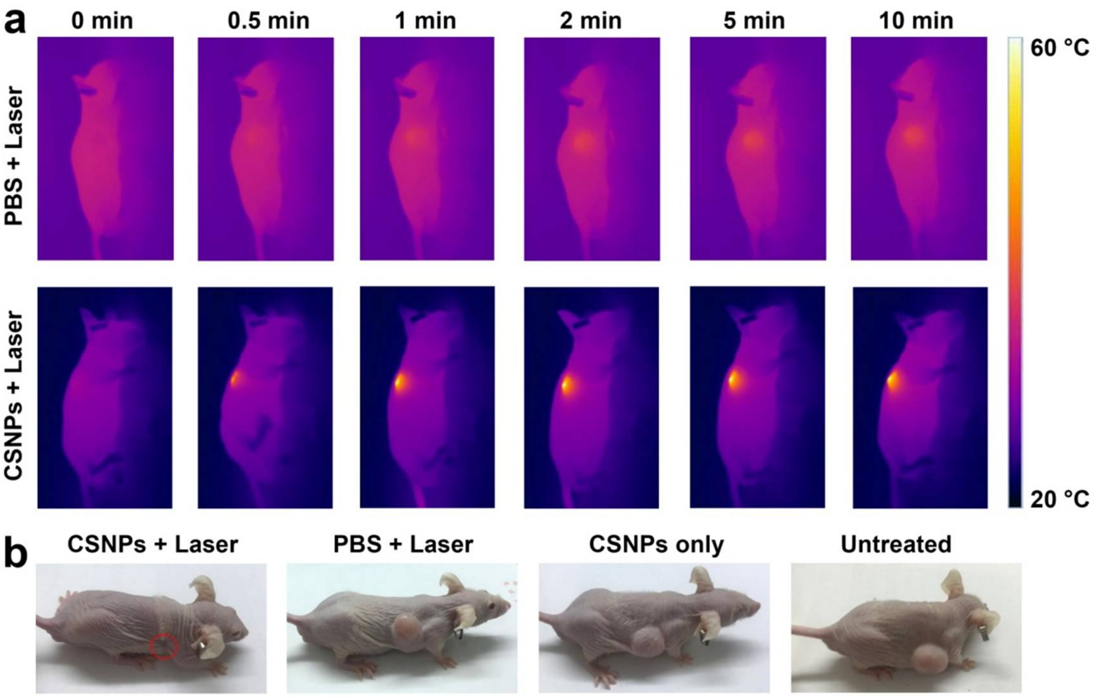

Figure S11. (a) IR thermal images of HNSCC mice with intratumour injection of PBS and $Cu_{2-x}S$@MnS CSNPs suspension under 808 nm laser irradiation taken at different time intervals in the PDX model. (b) Representative photos of mice collected on the 19th day after treatments.

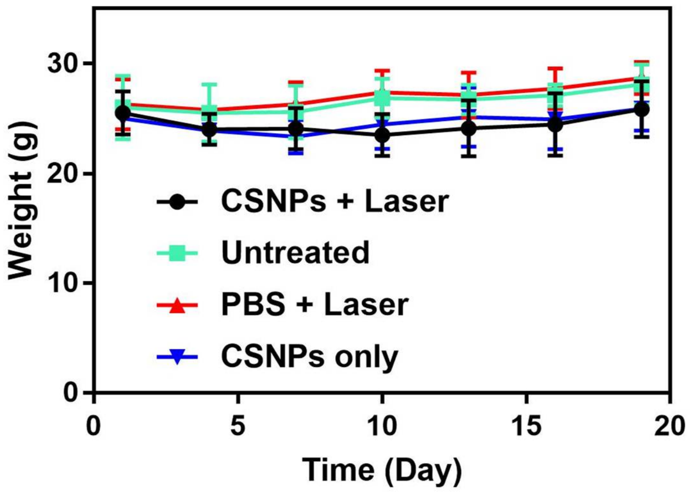

Figure S12. Average bodyweight curves of mice in different groups after various treatments in the PDX models.

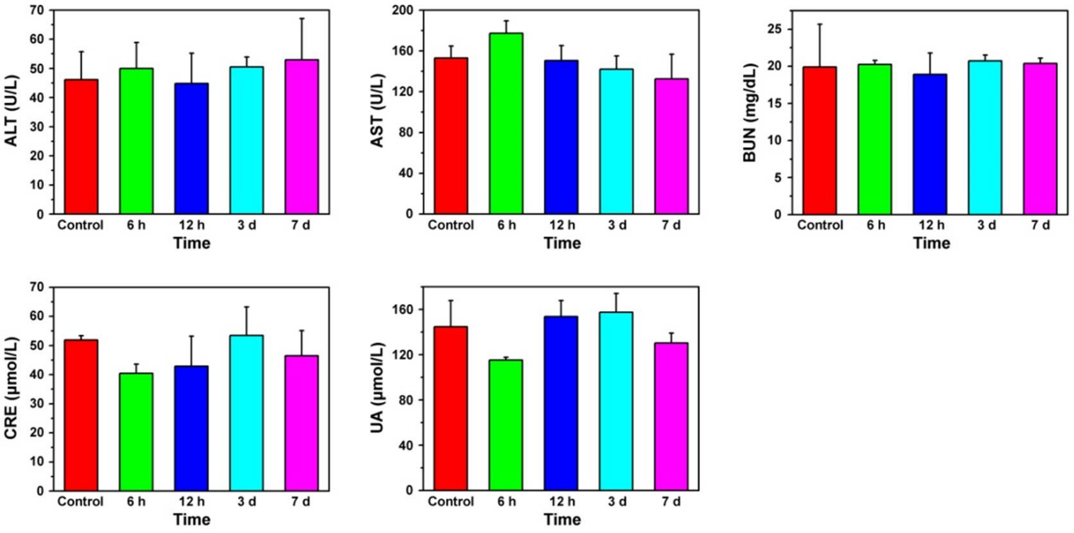

Figure S13. Blood biochemistry tests of mice with various treatments.

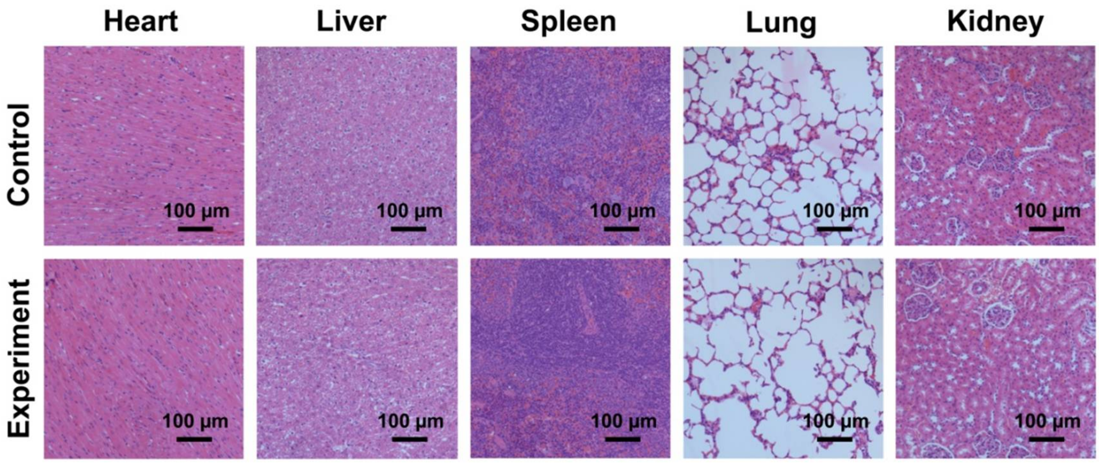

Figure S14. H&E-stained images of tissue sections from different organs of mice after $Cu_{2-x}S$@MnS CSNPs suspension injection (experiment) and healthy mice without treatment (control).

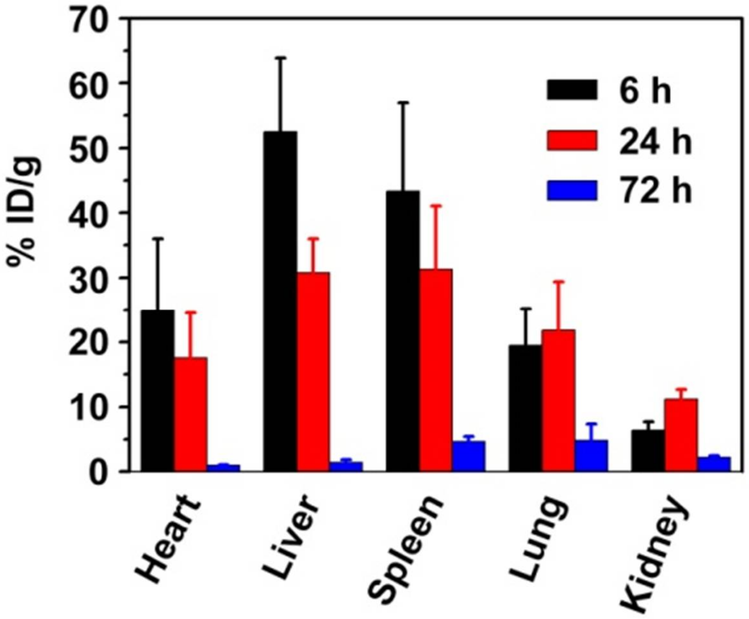

Figure S15. Tissue distributions of $Cu_{2-x}S$@MnS CSNPs post-injection.

References

[1] D. K. Roper, W. Ahn, M. Hoepfner, *J. Phys. Chem. C* **2007**, *111*, 3636.

[2] Q. Tian, F. Jiang, R. Zou, Q. Liu, Z. Chen, M. Zhu, S. Yang, J. Wang, J. Wang, J. Hu, *ACS Nano* **2011**, *5*, 9761.
